# Supplementary material for: S-acylation of P2K1 mediates extracellular ATP-induced immune signaling in Arabidopsis
Source: Nat Commun. 2021 May 12;12:2750. doi: 10.1038/s41467-021-22854-1 (PMC8115640; doi:10.1038/s41467-021-22854-1)
Supplement: Supplementary file 1 — Supplementary Information [file 41467_2021_22854_MOESM1_ESM.pdf]

Supplementary information

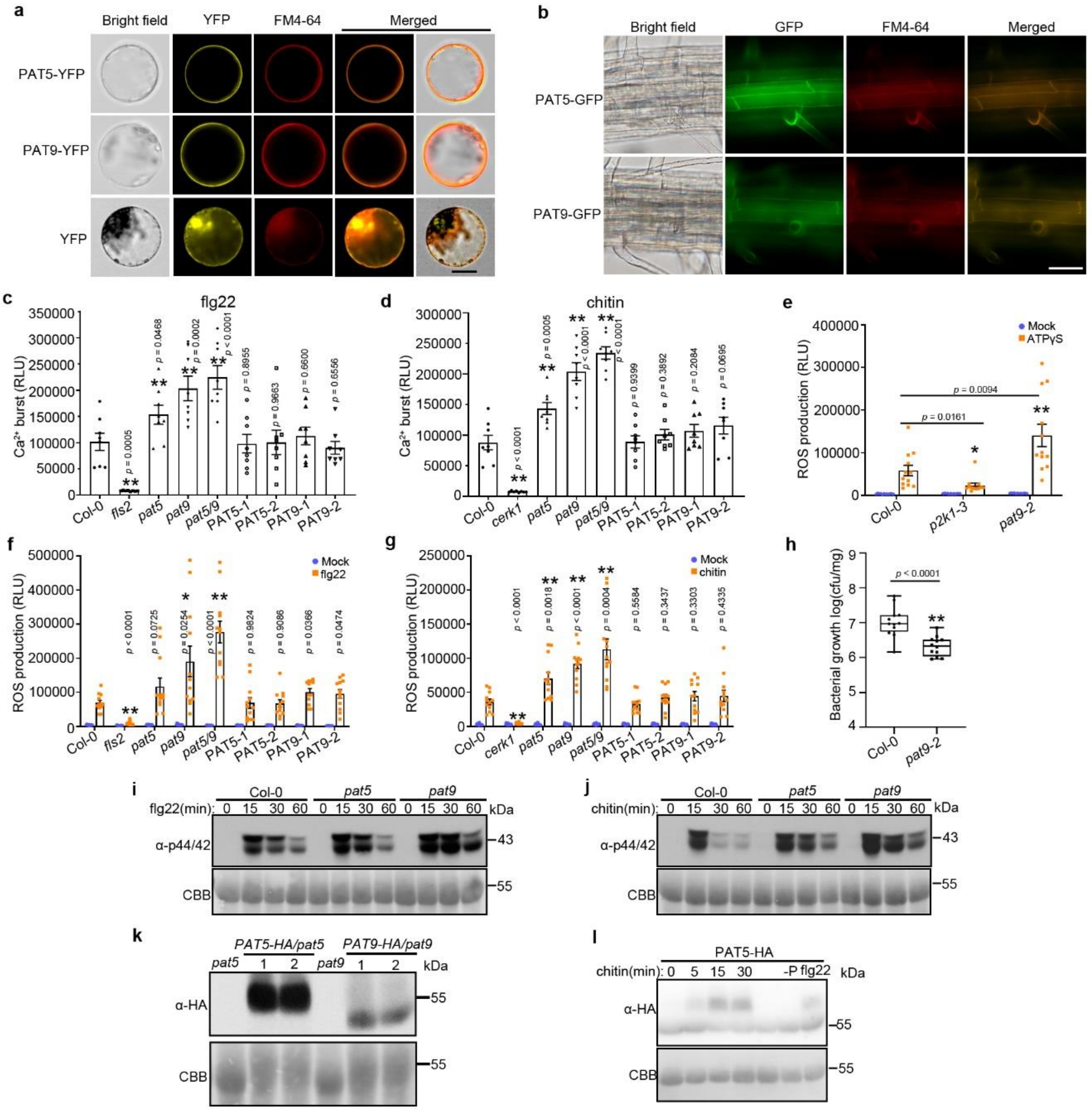

Supplementary Fig. 1. Subcellular localization of PAT5 and PAT9 and PAMP-induced PTI immune response

a PAT5-YFP and PAT9-YFP were transiently expressed in the *Arabidopsis* protoplasts, and merged well with the plasma membrane marker FM-64. Free YFP was used a control. Bar = 20 μm. **b** PAT5-GFP and PAT9-GFP were localized at plasma membrane in the roots of *Arabidopsis* stable transgenic plants. Bar = 50 μm. Experiments were repeated three times with similar results. **c-d** PAMPs-induced calcium influx. 5-day-old seedlings were treated with 1 μM flg22 or 50 μg/ml chitin for 30 min. RLU, relative luminescence units. Error bars indicate ± SEM; n = 8 seedlings; $*p < 0.05$, $**p < 0.01$, *P*-values indicate significance

relative to Col-0 and were determined by one-sided ANOVA with multiple comparisons and adjusted using Benjamini–Hochberg post-test. **e-g** ROS production was measured in leaf discs after treatment with 1 μM flg22 or 50 μg/ml chitin for 30 min. Leaf discs were taken from WT (Col-0), *pat5*, *pat9* and *pat5/9* double mutants, or their complemented lines *PAT5* (*NP::ATPAT5-HA/Atpat5*) and *PAT9* (*NP::ATPAT5-HA/Atpat5*). Error bars indicate ± SEM; n = 12 leaf discs; $*p < 0.05$, $**p < 0.01$, *P*-values indicate significance relative to Col-0 with ATPγS, flg22 or chitin treatment and were determined by one-sided ANOVA with multiple comparisons and adjusted using Benjamini–Hochberg post-test. **h** Bacterial invasion of *pat9-2* mutant. *Arabidopsis* seedlings were flood-inoculated with *Pst.* DC3000 bacteria and bacterial growth determined by plate counting 3 days post-inoculation (dpi). Error bars indicate ± SEM; n = 12 (biological replicates); $**p < 0.0001$ (two tailed), significance between Col-0 and *pat9-2* was determined by one-sided ANOVA with unpaired, two-tailed Student's *t* test. Box extends from the 25th to the 75th percentile, whiskers denote minima and maxima (Boxplots, Col-0: max = 7.763; min = 6.161; center = 6.975; Q2 (25%) = 6.747; Q3 (75%) = 7.214, *pat9-2*: max = 6.857; min = 5.954; center = 6.331; Q2 (25%) = 6.039; Q3 (75%) = 6.515). **i-j** MAPKs activation of *Arabidopsis* leaf discs that treated with 1 μM flg22 or 100 μg/ml chitin. Experiments were repeated three times with similar results. **k** Relative expression protein levels of *NP::ATPAT5-HA/Atpat5* and *NP::ATPAT9-HA/Atpat9* complemented transgenic lines using their own native promoters. CBB was used as a loading control. **l** Ligand triggers PAT5 phosphorylation after treated with 1 μM flg22 or 50 μg/ml chitin. Coomassie Brilliant Blue (CBB) staining of protein was used as loading control. Experiments were repeated three times with similar results.

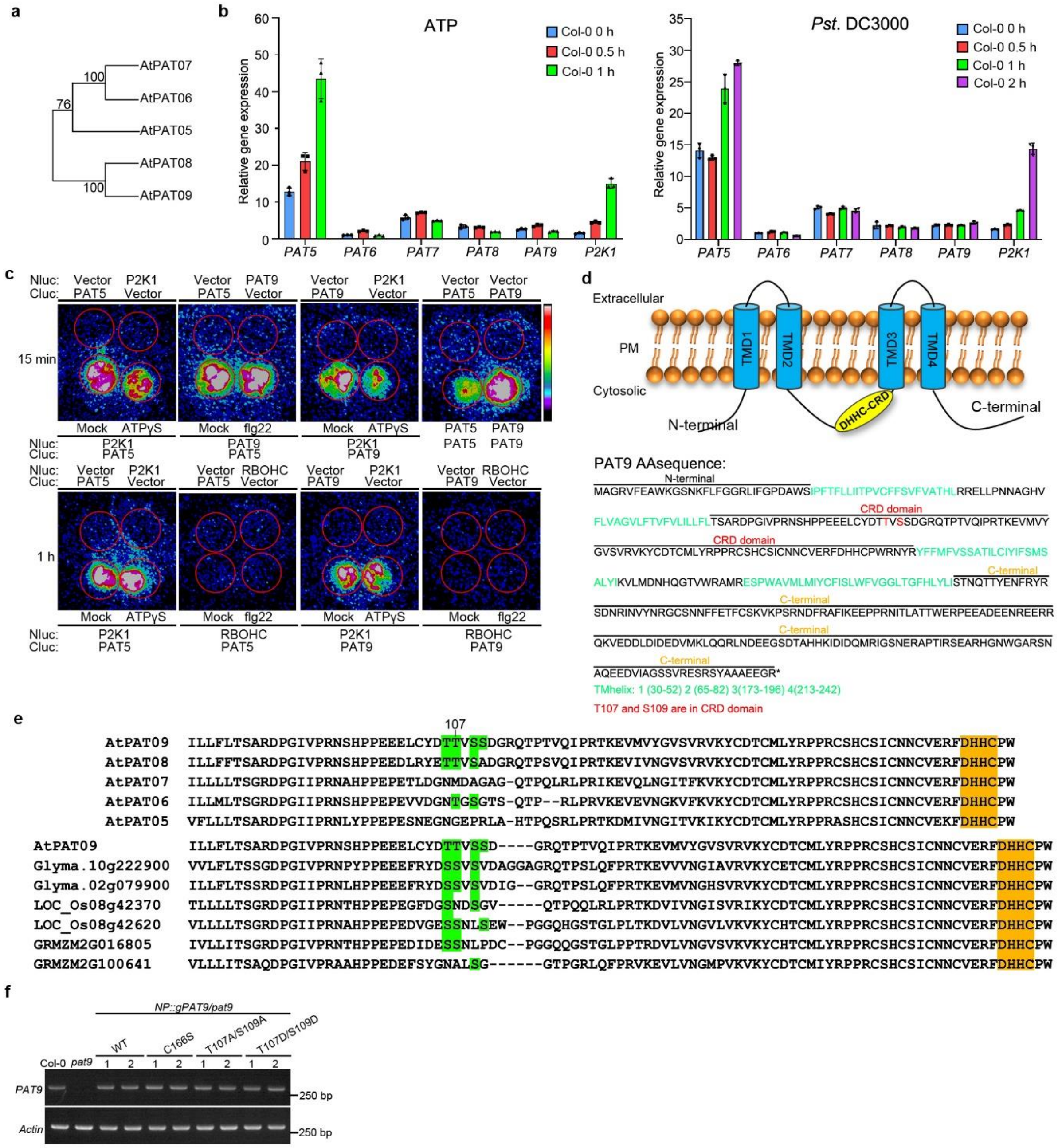

Supplementary Fig. 2. PAT5 and PAT9 interact with P2K1

a A simple phylogenetic tree of PAT5, PAT6, PAT7, PAT8 and PAT9 obtained by a maximum likelihood (ML) approach using the PALM pipeline through their amino acid sequences. **b** ATP and pathogen induced *PAT5-PAT9* and *P2K1* gene expression in wild-type (Col-0) plants. RNA was extracted from 4-5 weeks old leaves which infiltrated with 200 μM ATP and *Pst.* DC3000 (OD = 0.02). Error bars indicate ± SD; n = 3, biological replicates. **c** PAT5 and PAT9 interact with P2K1 in *N. benthamiana* in an ATP-dependent manner. The indicated constructs were transiently expressed in *N. benthamia* leaves with, where indicated in red circles, the

addition of 500 μM ATPγS. Nluc, N-terminal fragment of firefly luciferase; Cluc, C-terminal fragment of firefly luciferase; Vector, empty vector. Red circles, where the *Agrobacteria* was infiltrated and interaction happened. The color bar represents the color code for fluorescence intensities. **d** Protein topology of PATs on plasma membrane. PM, plasma membrane. TMD, transmembrane domains. DHHC, Asp-His-His-Cys. CRD, a stretch of DHHC within a Cys-rich domain. The transmembrane domains and T107/S109 were showed in PAT9 protein sequence. **e** Alignment part of AtPAT9 orthologs and paralogs proteins. The claimed phosphosites of PAT9 and DHHC domain are conserved in *Arabidopsis* and different species. **f** Relative of *PAT9* gene expression level in different transgenic plants. *PAT9* gene expression was detected by RT-PCR, *Actin* gene expression was used as a control. Experiments were repeated three times with similar results.

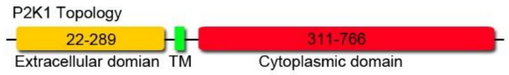

ID	Position	Peptide	Score	Cutoff	Cluster
P2K1	87	SFSTHFVCALVPKPG	0.447	0.0	S-Palmitoylation: Cluster B
P2K1	296	LIILLPVCLAILVLA	0.502	0.0	S-Palmitoylation: Cluster A
P2K1	394	AEVVSMRCLKHRNLV	2.301	1.991	S-Palmitoylation: Cluster B
P2K1	407	LVPLFGYCRRKRELL	1.966	0.903	S-Palmitoylation: Cluster C
P2K1	538	VFMLEVTCGRRPVEP	0.461	0.0	S-Palmitoylation: Cluster C
P2K1	559	RHMIKWVCECWKKDS	1.598	0.0	S-Palmitoylation: Cluster B
P2K1	561	MIKWVCECWKKDSLL	0.638	0.0	S-Palmitoylation: Cluster C
P2K1	594	VMKLGLLCSNIVPES	0.355	0.0	S-Palmitoylation: Cluster A

Supplementary Fig. 3. P2K1 protein domains and *S*-acylation sites prediction

Schematic representation of P2K1 protein structures contain an extracellular domain, a transmembrane domain (TM) and a cytoplasmic domain. The numbers in the topology and position indicate amino acids. The receptor protein sequences were analyzed by GPS-Lipid 1.0 software, the *S*-acylation residues were shown in red font.

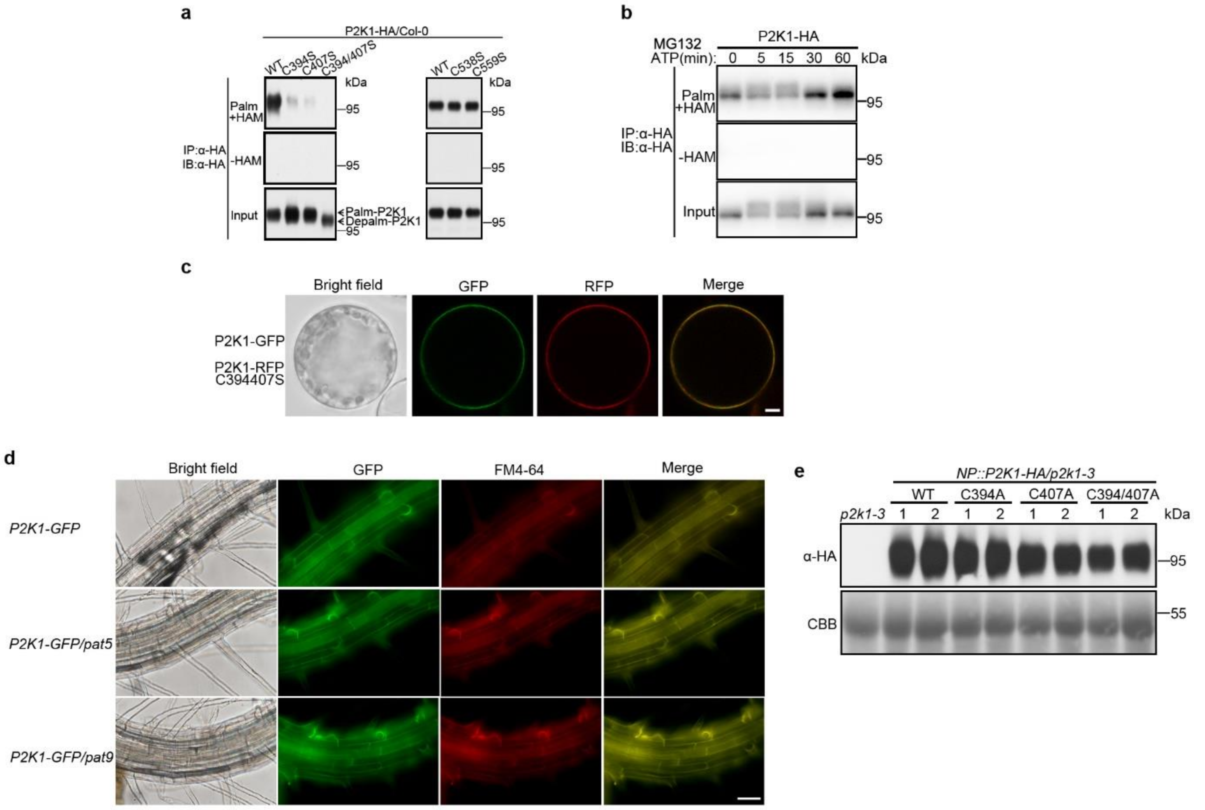

Supplementary Fig. 4. PAT5 and PAT9 regulate P2K1 *S*-acylation

a The P2K1 *S*-acylation residues were confirmed with C→S mutation in *planta*. The indicated constructs with HA tag were transiently expressed in *Arabidopsis* Col-0 protoplasts. The *S*-acylation levels were detected by an acyl-resin capture (acyl-RAC) assay. Palm-, palmitoylated protein; Depalm-, depalmitoylated protein. HAM, hydroxylamine, for cleavage of the Cys-palmitoyl thioester linkages. **b** Dynamic S-acylation levels of P2K1 in stable transgenic seedlings upon 200 μM ATP and protein degradation inhibitor. MG132 was used to inhibit receptor degradation. **c, d** The palmitoylation state of P2K1 does not affect their trafficking to the PM in protoplasts or roots of stable P2K1-GFP transgenic plants. P2K1-GFP was transient co-expressed with P2K1^{C394407S}-RFP in *Arabidopsis* protoplasts. The *35S:P2K1-GFP* plant was crossed with *pat5* and *pat9* mutant plants. Bar = 5 μM in **c**; bar = 50 μM in **d**. **e** Total P2K1-HA proteins were detected by anti-HA immunoblot for the stable transgenic plants. Full length of *P2K1* genomic DNA with about 1.5 kb native promoter was induced into *p2k1-3* mutant. CBB, Coomassie brilliant blue staining. All above experiments were repeated three times with similar results.

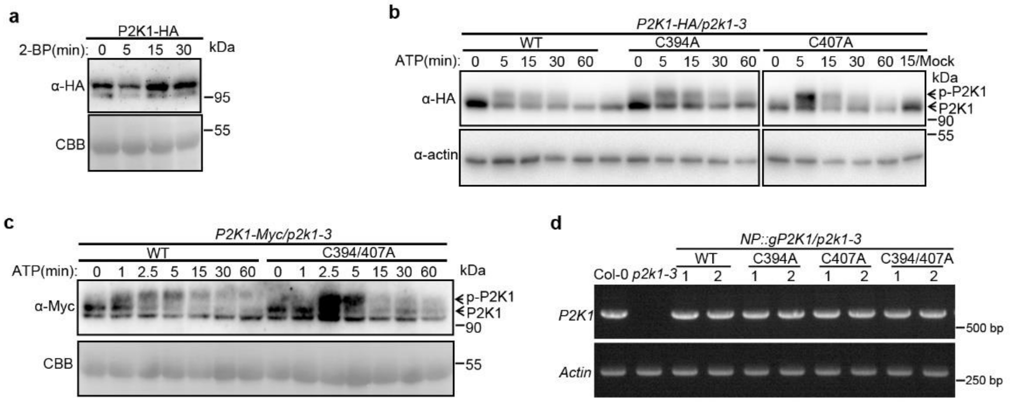

Supplementary Fig. 5. PAT5 and PAT9 regulate P2K1 phosphorylation and degradation

a P2K1-HA protein accumulation was analyzed by immunoblot in wild type plant upon addition of 50 μM 2-BP (*S*-acyltransferase inhibitor). **b** The ATP-induced P2K1 protein phosphorylation and protein degradation were detected in C394A and C407A mutated form. p-P2K1, phosphorylation of P2K1. **c** Another replicate of turnover and phosphorylation of P2K1 protein with modified (C→A) at the site of *S*-acylation. The indicated *Arabidopsis* plants were treated with 400 μM ATP for indicated time. CBB was used as a loading control. **d** *P2K1* gene expression in the indicated stable transgenic plants without any tag using RT-PCR. NP: native promoter. All above experiments were repeated and analyzed three times with similar results.

Supplementary Table 1. Sequence of primers used in this study

Primer	5'-3'sequence	Purpose
P2K1-B1	GGGGACAAGTTTGTACAAAAAAGCAGGCTTC ATGGCTCGTTGGTTGCTTCAGATC	For pDORN-Zeo
P2K1-B2	GGGGACCACTTTGTACAAGAAAGCTGGGTT CCTCTGACTGCTGATGCTGG	
P2K1pro-B1	GGGGACAAGTTTGTACAAAAAAGCAGGCTTC GAGAGGAGGTTCTTCCTGGTGg	
P2K1stop-B2	GGGGACCACTTTGTACAAGAAAGCTGGGTT GAAACAATCCCCAAGCCATtgct	
PAT5-B1	GGGGACAAGTTTGTACAAAAAAGCAGGCTTC ATGTTAGATTTGCAGCCGTCAG	
PAT5-B2	GGGGACCACTTTGTACAAGAAAGCTGGGTT TAACCGCCCTATACCAGTTCC	
PAT9-B1	GGGGACAAGTTTGTACAAAAAAGCAGGCTTC ATGGCTGGACGGGTCTTCGAA	
PAT9-B2	GGGGACCACTTTGTACAAGAAAGCTGGGTT CCGTCCCTCCTCTGCAGCTGCATA	
PAT9-ProB1	GGGGACAAGTTTGTACAAAAAAGCAGGCTTC AAGATGAGGTTACAAGCGCAGACC	
PAT9-B2gem	GGGGACCACTTTGTACAAGAAAGCTGGGTT AGGCAAAAACCAGAGTAACCT	
CD2b-B1	GGGGACAAGTTTGTACAAAAAAGCAGGCTTC ATGGCAGATAGTTCGTCGAGGA	
CD2b-B2	GGGGACCACTTTGTACAAGAAAGCTGGGTT AACTTCTTCAGTAGCAACTTCAG	
RBOHC-B1	GGGGACAAGTTTGTACAAAAAAGCAGGCTTC ATGTCTAGAGTGAGTTTTGAAG	
RBOHC-B2	GGGGACCACTTTGTACAAGAAAGCTGGGTT GAAATTCTCTTTGTGGAAGGAG	
P2K1-C394S-F	GTCGTGAGCATGAGATcTCTGAAACACAGGAATC	For site-directed mutagenesis
P2K1-C394S-R	GATTCCTGTGTTTCAGAgATCTCATGCTCACGAC	
P2K1-C407S-F	CCACTGTTTGGGTATTcCAGAAGAAAACGAGAAC	
P2K1-C407S-R	GTTCTCGTTTTCTTCTGgAATACCCAAACAGTGG	
P2K1-C538S-F	CATGCTTGAAGTAACCTcTGGGAGGAGACCCGTG	
P2K1-C538S-R	CACGGGTCTCCTCCCAgAGGTTACTTCAAGCATG	
P2K1-C559S-F	CATATGATCAAATGGGTTTcTGAGTGCTGGAAAAAGGATTCTTTGC	
P2K1-C559S-R	GCAAAGAATCCTTTTTCCAGCACTCAgAAACCCATTTGATCATATG	
P2K1-C394A-F	GTCGTGAGCATGAGAgcTCTGAAACACAGGAATC	
P2K1-C394A-R	GATTCCTGTGTTTCAGAgcTCTCATGCTCACGAC	
P2K1-C407A-F	CCACTGTTTGGGTATgcCAGAAGAAAACGAGAAC	
P2K1-C407A-R	GTTCTCGTTTTCTTCTGgcATACCCAAACAGTGG	
PAT5-C188S-F	GAAGTTTGATCACCACTcTCCTTGGCTTGGTCAG	
PAT5-C188S-R	CTGACCAAGCCAAGGAgAGTGGTGATCAAACTTC	
PAT9-C166S-F	GAGCGCTTTGATCATCATTcCCCTTGGAGAAACTATAG	
PAT9-C166S-R	CTATAGTTTCTCCAAGGGgAATGATGATCAAAGCGCTC	
PAT9-T107A/S109A-F	GAACTATGCTATGATACAgCTGTAgCAAGTGATGGAAGACAAACA	
PAT9-T107A/S109A -R	TGTTTGTCTTCCATCACTTGcTACAGcTGTATCATAGCATAGTTC	
PAT9-T107D/S109D-F	GAACTATGCTATGATACAgaTGTAgacAGTGATGGAAGACAAACA	
PAT9-T107D/S109D -R	TGTTTGTCTTCCATCACTgtcTACAtcTGTATCATAGCATAGTTC	
PAT5-CRD-F	TCGC GGATCC ACCTCCGCGAGAGACCCAGGGAT	For pET28a
PAT5-CRD-R	GTCA CTCGAG CCTATAGTTTCTCAATCCAATGC	
PAT5-C-F	TCGC GGATCC AGTACCAATCAGTCAACTTACG	
PAT5-C-R	GTCA CTCGAG TAACCGCCCTATACCAGTTCCG	
PAT9-CRD-F	TCGC GGATCC ACTTCTGCGCGGGATCCTGGAATC	

PAT9-CRD-R	GTCA CTCGAG CCTATAGTTTCTCCAAGGGCAATG	
PAT9-C-F	TCGC GGATCC AGCACAAACCAGACGACCTATGAG	
PAT9-C-R	GTCA CTCGAG CCGTCCCTCCTCTGCAGCTGCATAG	
CD2b-F	TCGC GAGCTC ATGGCAGATAGTTCGTCGAGGA	
CD2b-R	GTCA CTCGAG AACTTCTTCAGTAGCAACTTCAG	
Actin2-F	GCCATCCAAGCTGTTCTCTC	RT-PCR
Actin2-R	GCTCGTAGTCAACAGCAACAA	
UBQ-QF	TTCCTTGATGATGCTTGCTC	qRT-PCR
UBQ-QR	TTGACAGCTCTTGGGTGAAG	
P2K1-QF1	TGTTAGACGCGGAGTTCCAT	
P2K1-QR1	TACCCTACAGTCCCAACAGC	
PAT05-QF1	GCTGTTGCTGTTGGTCTGAT	
PAT05-QR1	GCAACCGACTCTGAGGAGTA	
PAT06-QF1	TGTTGCTGTTGTCTTCACCA	
PAT06-QR1	GGCAATCTTGGAGTTTGGCT	
PAT07-QF1	GCCGTCGTGTTCACCATTTA	
PAT07-QR1	GGTTTCAGGCTCTGGAGGAT	
PAT08-QF1	GATGCTAGGTCACTGCCTCT	
PAT08-QR1	CCCGCATTGTAGGGAGAGAA	
PAT09-QF1	CGCGAGCTCTTACCAAACAA	
PAT09-QR1	CTGGCGGATGCGAATTTCTT	
