# Supplementary material for: S-acylation of P2K1 mediates extracellular ATP-induced immune signaling in Arabidopsis
Source: Nat Commun. 2021 May 12;12:2750. doi: 10.1038/s41467-021-22854-1 (PMC8115640; doi:10.1038/s41467-021-22854-1)
Supplement: Supplementary file 3 — Reporting Summary [file 41467_2021_22854_MOESM3_ESM.pdf]

nature research

Corresponding author(s): Gary Stacey; Dongqin Chen

Last updated by author(s): Mar 21, 2021

Reporting Summary

Nature Research wishes to improve the reproducibility of the work that we publish. This form provides structure for consistency and transparency in reporting. For further information on Nature Research policies, see our Editorial Policies and the Editorial Policy Checklist.

Statistics

For all statistical analyses, confirm that the following items are present in the figure legend, table legend, main text, or Methods section.

n/a	Confirmed	
☐	☒	The exact sample size (n) for each experimental group/condition, given as a discrete number and unit of measurement
☐	☒	A statement on whether measurements were taken from distinct samples or whether the same sample was measured repeatedly
☐	☒	The statistical test(s) used AND whether they are one- or two-sided<br>*Only common tests should be described solely by name; describe more complex techniques in the Methods section.*
☒	☐	A description of all covariates tested
☐	☒	A description of any assumptions or corrections, such as tests of normality and adjustment for multiple comparisons
☐	☒	A full description of the statistical parameters including central tendency (e.g. means) or other basic estimates (e.g. regression coefficient) AND variation (e.g. standard deviation) or associated estimates of uncertainty (e.g. confidence intervals)
☐	☒	For null hypothesis testing, the test statistic (e.g. F, t, r) with confidence intervals, effect sizes, degrees of freedom and P value noted<br>*Give P values as exact values whenever suitable.*
☒	☐	For Bayesian analysis, information on the choice of priors and Markov chain Monte Carlo settings
☒	☐	For hierarchical and complex designs, identification of the appropriate level for tests and full reporting of outcomes
☒	☐	Estimates of effect sizes (e.g. Cohen's d, Pearson's r), indicating how they were calculated

Our web collection on statistics for biologists contains articles on many of the points above.

Software and code

Policy information about availability of computer code

Data collection	ROS, calcium and LCI data: Image32 (Photek, St Leonards on Sea, UK)<br>qRT-PCR data: ABI QuantStudio Software v1.3
Data analysis	S-acylation sites predict: GPS-Lipid 1.0 : http://lipid.biocuckoo.org/;<br>Western blots bands intensity: Image J;<br>Statistical analysis: GraphPad Prism 8.

For manuscripts utilizing custom algorithms or software that are central to the research but not yet described in published literature, software must be made available to editors and reviewers. We strongly encourage code deposition in a community repository (e.g. GitHub). See the Nature Research guidelines for submitting code & software for further information.

Data

Policy information about availability of data

All manuscripts must include a data availability statement. This statement should provide the following information, where applicable:

- Accession codes, unique identifiers, or web links for publicly available datasets
- A list of figures that have associated raw data
- A description of any restrictions on data availability

The authors declare that all raw data for this research are included in the paper and source data Excel file.

Field-specific reporting

Please select the one below that is the best fit for your research. If you are not sure, read the appropriate sections before making your selection.

- [x] Life sciences
- [ ] Behavioural & social sciences
- [ ] Ecological, evolutionary & environmental sciences

For a reference copy of the document with all sections, see nature.com/documents/nr-reporting-summary-flat.pdf

Life sciences study design

All studies must disclose on these points even when the disclosure is negative.

Sample size	Sample size was chosen as large as possible and in accordance with previous established protocols in the field. 5-day-old Arabidopsis seedlings were used for Calcium influx assay; leaf discs (about 0.55 cm diameter)were taken from 5-week-old plants were used for Oxidative (ROS) burst assay.
Data exclusions	no data were excluded from the analyses.
Replication	All experiments were successfully repeated at least 3 times, often containing multiple technical replicates.
Randomization	The seedlings or leaf discs are randomly selected or put together as a group.
Blinding	No blinding was utilized for our experiments.

Reporting for specific materials, systems and methods

We require information from authors about some types of materials, experimental systems and methods used in many studies. Here, indicate whether each material, system or method listed is relevant to your study. If you are not sure if a list item applies to your research, read the appropriate section before selecting a response.

Materials & experimental systems

n/a	Involved in the study	
☐	☒	Antibodies
☒	☐	Eukaryotic cell lines
☒	☐	Palaeontology and archaeology
☒	☐	Animals and other organisms
☒	☐	Human research participants
☒	☐	Clinical data
☒	☐	Dual use research of concern

Methods

n/a	Involved in the study	
☒	☐	ChIP-seq
☒	☐	Flow cytometry
☒	☐	MRI-based neuroimaging

Antibodies

Antibodies used	Anti-HA-Peroxidase: Roche, 12013819001; Monoclonal Anti-HA-Agarose: Sigma, A2095; Monoclonal Anti-MYC (clone 9E10): Sigma, SAB4700447; Anti-MYC HRP conjugate: Sigma, 16-213; Monoclonal Anti-polyHistidine: Sigma, H1029; GST-tag Antibody [HRP]: GenScript, A00130; Phospho-p44/42 MAPK (Erk1/2) (Thr202/Tyr204): Cell Signaling Tech, 9101.
Validation	Validation statements of commercial antibodies are available from manufacturers: Anti-HA-Peroxidase (https://www.sigmaaldrich.com/content/dam/sigma-aldrich/docs/Roche/Bulletin/1/12013819001bul.pdf); Monoclonal Anti-HA-Agarose (https://www.sigmaaldrich.com/content/dam/sigma-aldrich/docs/Sigma/Datasheet/6/a2095dat.pdf); Anti-MYC (https://www.sigmaaldrich.com/catalog/product/sigma/sab4700447?lang=zh&region=CN); Anti-MYC HRP (https://www.sigmaaldrich.com/catalog/product/mm/16213?lang=zh&region=CN); Anti-polyHistidine (https://www.sigmaaldrich.com/catalog/product/sigma/h1029?lang=zh&region=CN); GST-tag Antibody [HRP]: (https://www.genscript.com.cn/antibody/A00130_40-GST_tag_Antibody_HRP_pAb_Rabbit.html?src=Z886fNtkfOUx); p44/42 MAPK (Erk1/2) (https://www.cellsignal.com/products/primary-antibodies/phospho-p44-42-mapk-erk1-2-thr202-tyr204-antibody/9101)
